# Maturation-deficient chikungunya virus elicits protective immune responses in a murine challenge model

**DOI:** 10.1038/s41541-026-01478-w

**Published:** 2026-05-08

**Authors:** Danillo Lucas Alves Esposito, Jhefferson Barbosa Guimarães, Benedito Antonio Lopes da Fonseca, Beate Mareike Kümmerer

**Affiliations:** 1 Institute of Virology, Medical Faculty, University of Bonn, Bonn, Germany; 2 Department of Internal Medicine, School of Medicine of Ribeirao Preto, University of Sao Paulo, Sao Paulo, Brazil; 3 Department of Biomolecular Sciences, School of Pharmaceutical Sciences of Ribeirao Preto, University of Sao Paulo, Sao Paulo, Brazil; 4 German Centre for Infection Research (DZIF), partner site Bonn-Cologne, Bonn, Germany

**Keywords:** Biotechnology, Immunology, Microbiology

## Abstract

Maturation of chikungunya virus (CHIKV) particles is dependent on cleavage of the precursor glycoprotein p62 into the proteins E3 and E2 by the cellular protease furin. Here, we produced immature CHIKV particles by infecting furin-deficient cells and characterized them in comparison to mature particles by electron microscopy. Ectopic expression of furin in the furin-deficient cells restored the production of infectious particles, underpinning the importance of this enzyme in particle maturation. To prevent furin‑mediated maturation in mammalian cells, we engineered a recombinant CHIKV in which the furin cleavage site was replaced by a Tobacco Etch virus (TEV) protease site, rendering the virus non‑infectious unless treated in vitro with TEV protease. TEV‑matured particles became infectious and, when used for immunization, induced a stronger immune response than their untreated counterparts, as evidenced by higher neutralizing antibody titers. In vivo, a single immunization with TEV‑treated particles protected IFNAR‑/‑ mice against a lethal CHIKV challenge. In immunocompetent mice, vaccination also reduced viremia and CHIKV‑induced footpad swelling. Together, these findings demonstrate that in vitro maturation of CHIKV modified with a TEV cleavage site promotes a safe, replication limited immunogenic particle, establishing a promising platform for developing vaccines against viruses that require furin protease maturation.

## Introduction

Chikungunya fever is caused by an alphavirus (chikungunya virus, CHIKV - *Alphavirus chikungunya*) of the *Togaviridae* family, typically found in tropical and subtropical climates. The word chikungunya comes from the Makonde dialect, meaning “that which bends up” or “bent walking,” referring to the posture of patients as a result of the intense arthralgia/arthritis that follows infection with this virus^1^. The virus was first reported in 1952 in Tanzania and is transmitted by mosquitoes of the genus *Aedes*, with a wild cycle involving non-human primates and/or large mammals, and an urban cycle where humans serve as hosts^2^.

After infection and an incubation period of 2–4 days the symptomatic phase of the disease follows. It is characterized by high fever (above 39 °C), myalgia, conjunctivitis, joint edema, non-pruritic maculopapular rash, and arthralgia or arthritis (present in almost 90% of patients and characteristic of this disease), which may persist for weeks or even months^3^. Symptoms may differ from one patient to another, but fever and joint inflammation are present in most cases and could be used as a differential diagnostic from other arboviral infections. During the acute phase of the disease, a short viremia, lasting about 2 to 4 days, is universally present and used to confirm the etiology of the disease^1^.

CHIKV infections remain a major scientific and public health challenge, as no antiviral treatment is currently available. Currently, two chikungunya vaccines are available: one live-attenuated vaccine (IXCHIQ) and one virus-like particle vaccine (VIMKUNYA)^4^. Although the live-attenuated Valneva vaccine IXCHIQ® was approved following Phase III trials, demonstrating robust neutralizing antibody responses^5^, the emergence of significant safety concerns has altered its use in older populations. In May 2025, regulatory agencies, including the FDA and CDC recommended pausing vaccinations in individuals aged ≥60 due to 17 serious adverse events reported globally in recipients aged 62–89, including two fatal events, possibly linked to the vaccine^6^. The EMA (European Medicines Agency) and UK MHRA (Medicines and Healthcare products Regulatory Agency) also suspended use in adults ≥65, based on the same safety data^7^. In Brazil, the National Health Surveillance Agency (ANVISA) approved the IXCHIQ® vaccine for adults aged 18 and above in April 2025, making it the first chikungunya vaccine authorized in an endemic country. However, its implementation requires additional efficacy and safety data within local epidemiological contexts^8^. These findings suggest that the live-attenuated Valneva vaccine may not be safe for elderly individuals or those with immunosuppression or underlying comorbidities, highlighting the need for continued development of safer vaccine alternatives for vulnerable populations.

In contrast, the virus‑like particle vaccine VIMKUNYA has demonstrated a favorable safety profile and strong seroconversion rates across children, adolescents, and adults^9,10^ Although no severe adverse effects, hospitalizations, or deaths were reported in post-approval vaccine trials, more comprehensive monitoring is necessary to expand vaccine use, which is currently recommended mainly for travelers heading to areas where chikungunya transmission may occur, as well as for laboratory personnel who might come into contact with the virus^11^.

The positive-stranded RNA genome of CHIKV is nearly 12 kilobases in length and contains two open reading frames (ORFs) flanked by 5′ and 3′ untranslated regions (UTRs). While the nonstructural proteins (nsP1-4) are directly translated from the 5′ two-thirds of the genome encompassing the 5′ORF, the structural proteins (C-capsid, E3, E2, 6K, and E1) are translated from a subgenomic RNA^12^. Infection of susceptible cells initiates with virus penetration through the interaction of cellular receptors and viral envelope proteins (E2 and E1), followed by clathrin-mediated endocytosis^13^. Within the endosome, acidification occurs, and thereby, the E2-E1 dimer disintegrates, exposing the fusion loop located in the distal region of the E1 protein. This results in the fusion of the viral membranes and the endosome, releasing the capsid into the cytoplasm, which disassembles, releasing the viral RNA into the cytoplasm. Translation of the viral RNA occurs in two steps: first, the non‑structural proteins are translated and assemble into the early replicase complex; this complex then synthesizes a negative‑strand RNA from the positive‑strand genome, which serves as the template for both genomic and subgenomic RNA synthesis. The latter will serve as a template for genome replication and production of subgenomic RNA that will be translated into the structural proteins. The structural proteins are formed in membranous structures of the cytoplasm (viral factories), where the capsid assembly occurs, and maturation of envelope proteins occurs in the endoplasmic reticulum and Golgi complex, which are exported to the cell membrane. New viral particles are formed by budding from the host cell membrane^14^.

Immature alphaviruses are particles that did not undergo specific cleavage by the furin protease during viral protein transport through the Golgi apparatus. Furin is a host protease that cleaves the viral glycoprotein precursor p62 (E3-E2) into the active form of E3 and E2 proteins under acidic conditions. This process usually takes place in the trans-Golgi vesicles, but it could also occur in the early stage of the endosome^15^. Most studies on immature particles were conducted with flaviviruses, especially the dengue virus (DENV), but some investigations using the alphaviruses Sindbis and chikungunya have shown that cleavage of the p62 precursor is required for infectivity^16,17^, and that the use of furin inhibitors can block viral spread in cell culture^18^.

It has been shown that immature or partially mature particles of DENV, when transported to the interior of the cells, undergo a maturation process under the low pH in the endosome and the action of furin. This process culminates in the cleavage of the DENV “*pr*” peptide of the M protein, enabling the fusion between the virus envelope protein and the endosome membrane^19^. The DENV “pr” peptide elicits an antibody-mediated immune response, resulting in antibodies that bind to immature viral particles. However, these antibodies have poor neutralizing capacity and facilitate an alternative viral entry pathway by interacting via their Fc region with cellular Fc receptors^19,20^. This may lead to an increase in virus entry into the cell, aggravating the disease, a phenomenon known as Antibody-Dependent Enhancement^21^. It has been shown that immature or partially mature DENV particles can influence the progression of the disease, both in patients infected for the first time and in heterotypic infections of DENV, increasing the severity of symptoms^22^.

The infectious potential of immature CHIKV particles remains largely unexplored. Although the presence of the E3 protein does not change the conformational structure of the virus particles^14^, and some alphaviruses appear to bud from the cell membrane with the E3 bound on its surface^23^, it has been shown that a full cleavage of this protein is necessary for viral particles to become fully infectious^24^. Further, this cleavage may occur either in the trans-Golgi or in the endosome vesicles. Thus, molecular patterns of infection by alphaviruses still need to be elucidated. Understanding the role of immature particles in those infections could represent a pivotal point in the treatment and prevention of new infections.

This study aimed to determine certain infectious properties of immature CHIKV particles by using a recombinant CHIKV virus in which the furin cleavage site between E3 and E2 was replaced by a cleavage site of another protease to assess whether suppression of virus maturation could lead to non-infectious immature CHIKV particles. These non-infectious particles were tested for vaccine development, as their inability to revert to a mature, infectious form reduces the risk of disease while still allowing the induction of a protective immune response.

## Results

### Production of immature particles in LoVo cells

LoVo cells, deficient in active furin protease due to a stop codon in the protein-coding sequence, were used to produce immature particles. CHIKV BzH1 was used to infect these cells, and the resulting non-infectious immature particles were quantified as genome-containing particles (GCP)/mL using RT-qPCR. After ultracentrifugation and storage at −80 °C, the titer of the immature particles reached 5 × 10⁵ GCP/mL. By comparison, mature CHIKV BzH1 produced in Vero cells had a titer of 3 × 10⁶ GCP/mL or 2.5 × 10⁵ PFU/mL, and recombinant CHIKV particles from infectious clones reached approximately 10⁶ GCP/mL.

### Characterization of immature particles and infectivity tests

**Fig. 1:**
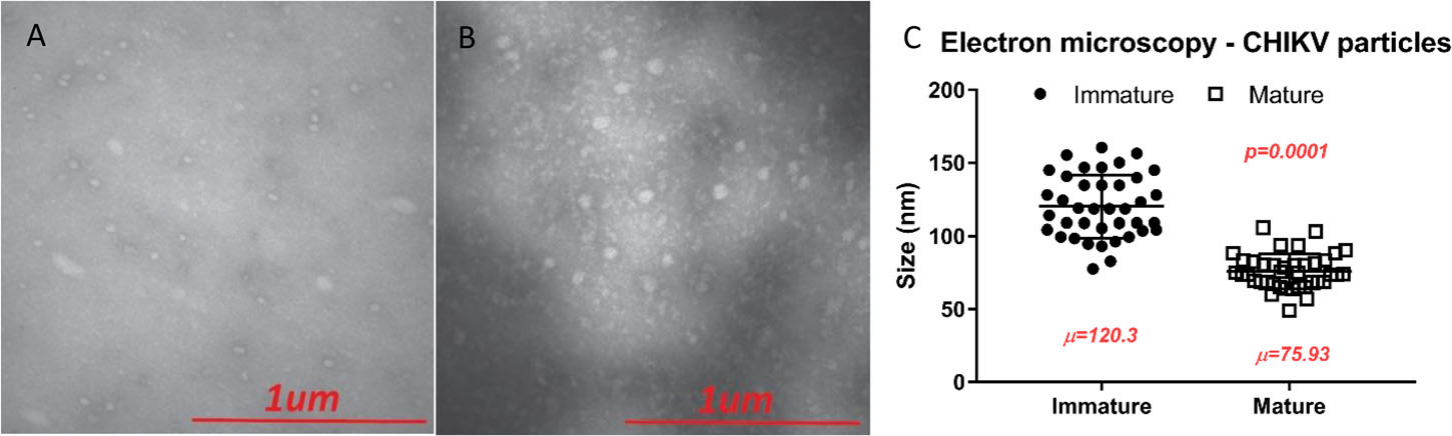
Structural comparison between immature and mature CHIKV particles. Transmission electron microscopy reveals morphological differences between immature (**A**) and mature (**B**) chikungunya virus (CHIKV) particles visualized by negative staining. **C** The average diameter (in nanometers) of immature and mature particles, measured from multiple fields. Data are presented as mean ± SD from three independent experiments. Student’s *t*-test was used to assess statistical differences between groups (*p* = 0.0001).

Immature particles (produced in LoVo cells) and mature particles (produced in Vero cells) were visualized by electron microscopy. Although the resolution of the equipment available at the time limited detailed morphological assessment, it was still possible to measure particle diameters reliably. Approximately 100 particles from each preparation were measured. Mature particles showed a mean diameter of 75.93 ± 11.74 nm, consistent with values reported in the literature (around 70 nm), whereas immature particles had a mean diameter of 120.3 ± 21.45 nm (Fig. 1A–C). A *t*‑test indicated that the size difference between immature and mature particles was statistically significant (*p* = 0.0001, *t* = 11.28).

**Fig. 2:**
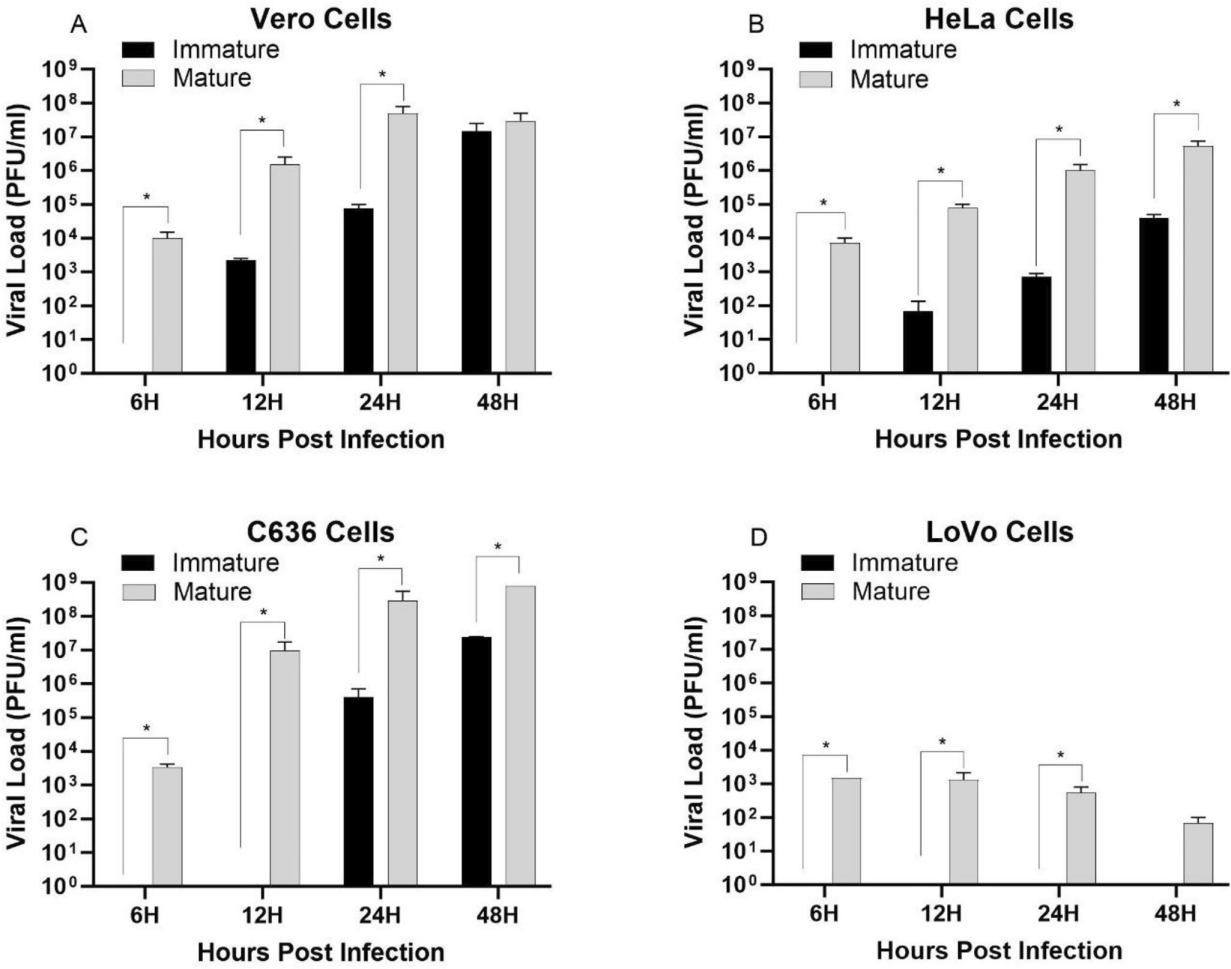
In vitro infectivity of immature particles. Infectivity and replication of CHIKV immature particles in mammalian (**A**–Vero, **B**–HeLa, **D**–LoVo) and insect (**C**–C6/36) cells. Cells were infected at an MOI of 0.1 and viral titers quantified by plaque assay at the indicated time points. Data represent mean ± SD of three independent experiments. Statistical analysis was performed on Log(Y + 1) transformed data using two-way ANOVA followed by Sidak’s multiple comparison test (^*^p < 0.05).

In infectivity tests, immature particles produced in LoVo cells exhibited a delay in viral progeny production in furin-competent cells (Vero, C63/36, and HeLa), eventually reaching titers comparable to mature particles (Fig. 2A–C). In LoVo cells, no viral progeny was observed following infection with immature particles, while mature particles produced reduced titers (Fig. 2D).

**Fig. 3:**
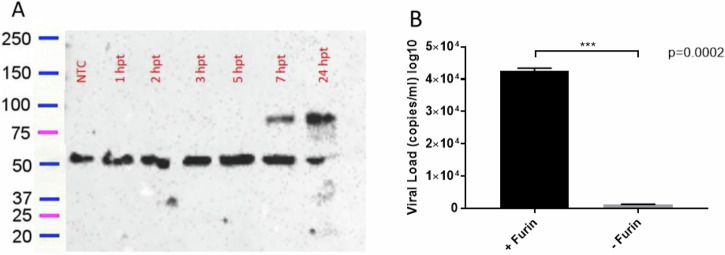
CHIKV viral load in LoVo cells after transient transfection with a plasmid expressing functional furin gene. **A** A time-course Western blot demonstrates the expression of furin protein at various time points post-transfection (hpt – hours post-transfection) (upper band); **B** immature virus was able to recover infectivity 24 h post-infection in LoVo cells after plasmid transfection (+Furin). Data represent mean ± SD from triplicate experiments. Statistical significance was assessed using an unpaired Student’s *t*-test (*p* = 0.0002). NTC not transfected cells.

To confirm the importance of furin for the infection of immature particles, a plasmid encoding the entire protease sequence was transfected in LoVo cells, restoring the furin protease expression within this cell line. The LoVo cell has a mutation in the furin gene that produces a non-functional 57 kDa protein due to the insertion of a stop codon in the protein sequence, which is usually about 87 kDa. After 8 h of transfection, full-size furin protease could be detected in LoVo cells in addition to the truncated version (Fig. 3A), and infection with immature particles was performed. A significant increase in produced CHIKV particles (*p* = 0.0002, Fig. 3B) was observed compared to untransfected cells.

### Replacement of furin cleavage site with TEV cleavage site

To analyze whether immature particles represent a useful tool for vaccination, the sequence encoding the furin cleavage site in the CHIKV cDNA clone was replaced by the sequence of another protease, namely the Tobacco Etch virus (TEV) protease. This protease is not present in mammalian or insect cells, and thus, it would prevent the particles’ natural maturation, but allow their in vitro maturation in the presence of a recombinant TEV protease. The respective virus (CHIKV-TEV) was produced using a previously described CHIKV infectious cDNA clone. For easier follow-up in initial cell culture experiments, we also established a TEV cleavage site encoding CHIKV cDNA clone expressing an mCherry red fluorescent protein incorporated within the viral nsP3 gene (nsP3-490) (CHIKV-TEV with mCherry)^25^.

After confirming the replacement of the sequence encoding the furin cleavage site by sequencing, RNAs in vitro transcribed from the CHIKV-TEV and CHIKV-TEV mCherry cDNA clones were electroporated into BHK-21 cells. RNA derived from the wild-type CHIKV-mCherry cDNA clone served as a positive control. For the mCherry expressing viruses, successful replication could be demonstrated via immunofluorescence analyzes at 24 h post electroporation (Supplementary Fig. 1). In addition, the positive control (CHIKV mCherry) showed an intense cytopathic effect on cells, as could be seen by plaque formation in an infectious center assay, while no visible cytopathic effect was observed in the cells electroporated with CHIKV-TEV mCherry RNA (Supplementary Fig. 1).

**Fig. 4:**
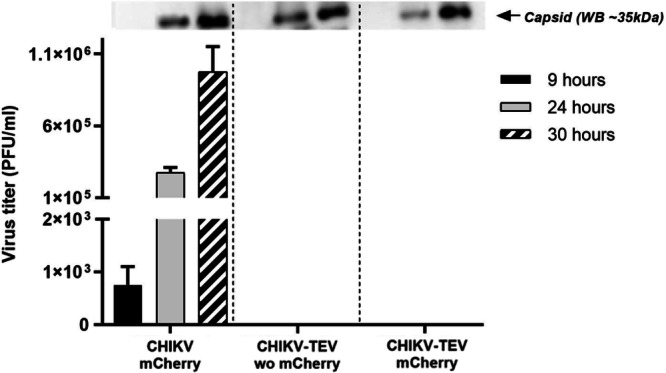
Virus production after electroporation in BHK-21 cells. Bottom image: Virus titer measured by plaque assay in Vero cells. Top – Western blot from the supernatant, detecting the production of particles (by anti-capsid antibody) for all viruses produced. Wo = without.

Further, at times 9, 24 and 30 h after electroporation, the cells were analyzed for production of virus particles in the supernatant (Fig. 4). To determine viral titers, the supernatants from all time points were assayed by a plaque assay using Vero cells. While the CHIKV mCherry positive control produced infectious virus with a significant increase of viral titers over time, no infectious particles were observed for CHIKV-TEV (Fig. 4 – Bottom).

To ensure that CHIKV-TEV particles were also secreted by the cells despite the lack of plaque formation, detection of the capsid protein from the supernatant was carried out by Western blot analyzes. All viruses yielded a positive band at 24 and 30 h after electroporation, but no band was detected after 9 h, probably due to the low amount of virus produced in this period (Fig. 4 - Top). Thus, it could be demonstrated that, despite no cytopathic effect was observed, particles were released into the supernatant consistent with the production of immature particles.

To confirm that the absence of viral plaque formation was due to the lack of furin cleavage, and not the slightly larger size of the TEV cleavage site, the furin cleavage sequence (RQRR) was mutated by site-directed mutagenesis to a sequence not recognized by furin protease (SQRS). This mutated clone showed the same inability to form plaques as the immature particles with the TEV cleavage site replacement (Supplementary Fig. 1).

### In vitro cleavage of TEV protease and cell infection

**Fig. 5:**
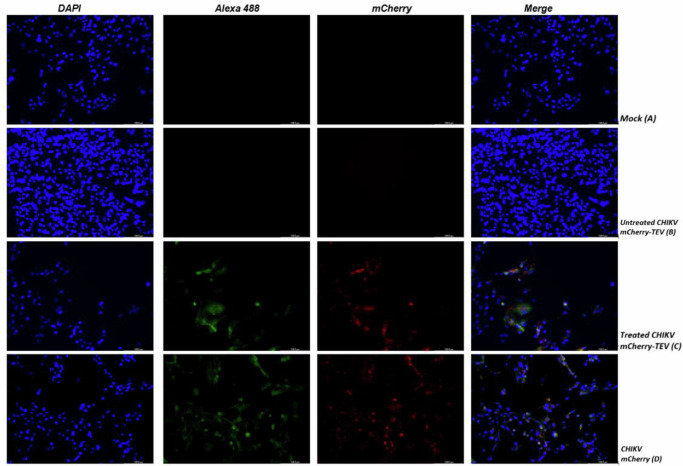
BHK-21 cells infection (FIRST INFECTION) with immature CHIKV particles. Mock (**A**); untreated CHIKV-TEV (**B**); TEV treated CHIKV-TEV (**C**) and CHIKV mCherry (**D** – positive control). Images were taken after 24 h of infection with M.O.I of 1. Green channel shows nsP1 protein, Red channel mCherry associated with nsP3 and blue channel, cell nucleus.

**Fig. 6:**
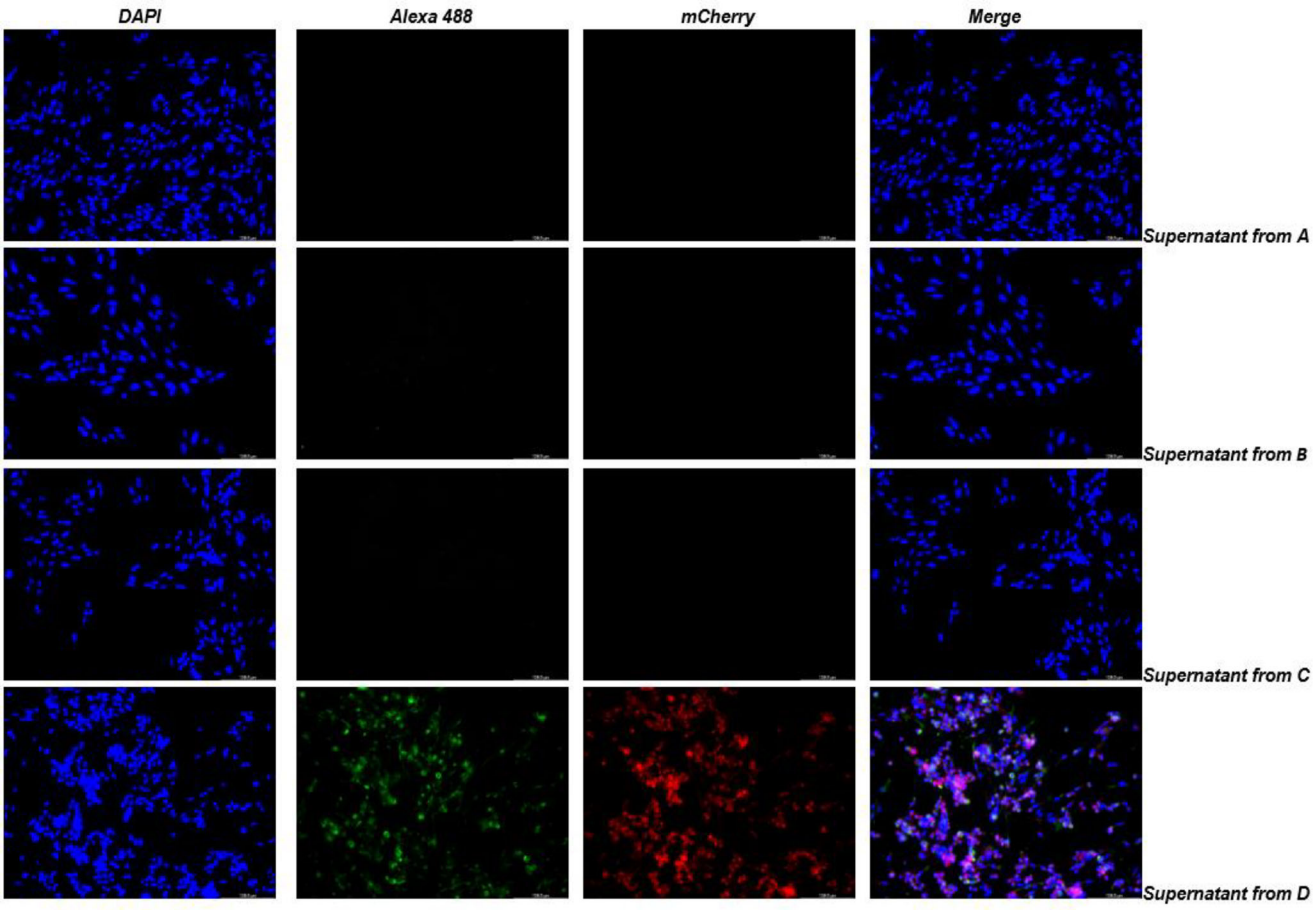
BHK-21 cells infected (SECOND INFECTION) with the supernatant of FIRST INFECTION. Mock (**A**); untreated CHIKV-TEV (**B**); treated CHIKV-TEV (**C**) and CHIKV mCherry (**D** – positive control). Images were taken after 24 h of infection with 250 µL of first infection supernatant. Green channel shows nsP1 protein, Red channel mCherry associated with nsP3 and blue channel, cell nucleus.

**Fig. 7:**
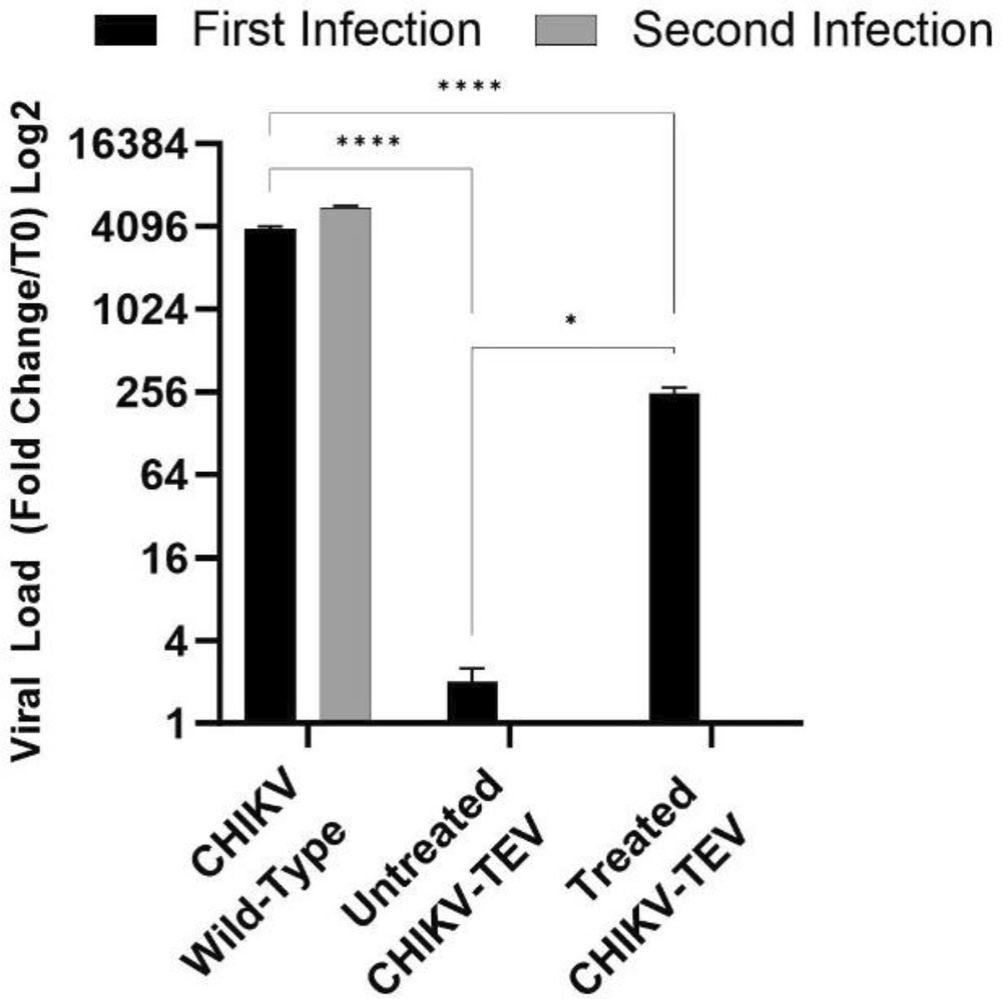
CHIKV viral load, measured by RT-qPCR from supernatant of infected BHK-21 cells, with or without prior treatment with TEV protease. First infection was made with immature particles, one batch with TEV protease treatment (treated CHIKV-TEV) and another without treatment (untreated CHIKV-TEV). The second infection was made with the supernatant from the first infection within each group. A wild-type virus was used as a positive control. Data represent mean ± SD from triplicate experiments. Statistical significance was assessed using Two-Way ANOVA test (^*^p < 0.05 and ^****^p < 0.0001).

To verify whether particles containing the TEV cleavage site are capable of infection after in vitro cleavage and reinfection, experiments were carried out in BHK-21 cells with and without prior in vitro incubation with TEV protease (treated and untreated with TEV in Figs. 5–7). Uninfected cells were used as a negative control and a wild-type CHIKV expressing mCherry as a positive control. No infection could be detected when untreated CHIKV-TEV mCherry virus was added to the cells (BHK-21), measured by mCherry signal production, intracellular nsP1 production (Fig. 5), and virus secreted in the supernatant (Fig. 7). After incubation with TEV protease, CHIKV-TEV mCherry restored its infection potential (Figs. 5 and 7).

The supernatant from the previous experiment was then subjected to reinfection (second infection) of the BHK-21 cells to see if the viral progeny could infect the cells or if it was utterly dependent on TEV protease treatment. As shown in Figs. 6 and 7, no infection was detected for the CHIKV-TEV virus, confirming that infection could only occur with particles that were previously treated in vitro with TEV protease.

To further evaluate the safety of the CHIKV-TEV particle, five sequential passages were performed using virus that was either pre‑treated or not treated with TEV protease. Consistent with previous findings, the TEV‑treated virus generated detectable infectious particles only in the first passage, with no plaques observed in the four subsequent rounds. In contrast, the untreated virus remained non‑infectious throughout all five passages in both C6/36 and Vero cells. These data confirm that neither condition restored replication competence with consecutive passages (Supplementary Fig. 2).

### Evaluation of the protective effect of the inactive CHIKV vaccine composed by immature particles

**Fig. 8:**
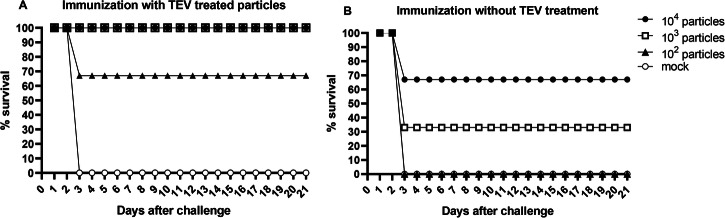
Survival of IFNAR mice after immunization with immature CHIKV particles. **A**, **B** The survival rate of IFNAR mice after immunization with immature particles (with TEV pre-treatment - **A**, and without TEV treatment – **B**, *n* = 10 per group) and challenge with wild-type virus.

After confirming that the produced immature CHIKV particles were incapable of initiating infection beyond the first replication cycle, vaccine protection experiments were conducted in mice. Immature particles were either cleaved in vitro using recombinant TEV protease (+TEV, Fig. 8A) or left uncleaved (−TEV, Fig. 8B), and both forms were used to immunize interferon receptor-deficient mice (IFNAR⁻/⁻). The mice received 10², 10³, or 10⁴ particles of either preparation. Upon challenge with a wild-type CHIKV strain (BzH1), all animals receiving 10^4^ or 10^3^+TEV treated particles survived, and only three animals from the +TEV group receiving the lowest dose of 10^2^ particles succumbed to the infection (representing 10% of all mice inoculated with pre-treated particles). In contrast, animals died in all groups when mice where immunized with uncleaved particles (-TEV). While 7 or 3 out of 10 mice survived in the 10^4^ or 10^3^ particle group, respectively, all mice that received only 10^2^ particles died. Hence, overall, approximately 67% of the mice immunized with uncleaved particles (−TEV) died by day 3 post-challenge. All animals in the mock-immunized group showed clinical signs of infection and died on the third day (Fig. 8A, B).

**Fig. 9:**
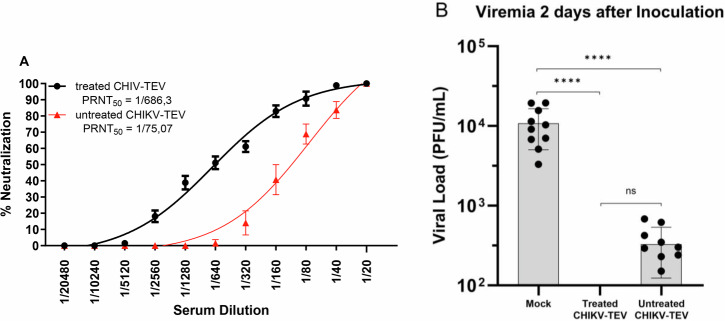
Antibody responses and post‑challenge viremia in vaccinated C57BL/6 mice. **A**, **B** Serological profile of vaccinated C57BL/6 mice. **A** Antibody production in C57BL/6 mice (*n* = 10 per group). CHIKV neutralizing antibodies were determined via plaque reduction assay. **B** Viremia after challenge in vaccinated mice, measured by plaque formation assay in blood samples collected 2 days post-challenge. Treated CHIKV‑TEV-vaccinated mice showed no detectable viremia, while 9 out of 10 untreated CHIKV‑TEV-vaccinated animals displayed measurable viral loads. Mock animals presented significantly higher viremia compared to both vaccine groups (^****^p < 0.0001). No statistical difference was observed between treated and untreated CHIKV‑TEV groups (ns). Each dot represents an individual animal; bars indicate median values. Statistical significance was assessed using Two-Way ANOVA test and Tukey’s multiple comparison test.

To further evaluate the efficacy of the vaccination protocol and assess the induction of neutralizing antibodies, wild-type C57BL/6 mice were immunized with either in vitro TEV-treated particles or untreated particles. Each group received one-dose immunization of 10^3^. Serum samples were collected 21 days after immunization to measure CHIKV neutralizing antibody levels. Mice immunized with TEV-treated particles showed approximately 9.14-fold higher neutralization capacity, as determined by PRNT₅₀ values, compared to those immunized with untreated particles (*P* = 0.0002, paired *t*-test; Fig. 9A). Taken together, the data show an improved survival rate and higher production of neutralizing antibodies when immunizations were performed with TEV-treated immature particles.

Two days after challenge with 10⁶ copies of wild‑type CHIKV (BzH1), mock‑immunized mice displayed high viremia (Fig. 9B), with values consistently around 10⁴ PFU/ml. Animals immunized with the treated CHIKV‑TEV particles showed no detectable viremia after challenge. In contrast, mice that received the untreated particles still displayed measurable viral loads, with viremia detected in 9 out of 10 animals (the non-viremic animal is not plotted due to log scale limitations). Both CHIKV‑TEV groups had significantly reduced viremia compared with the mock group (^**^p < 0.0001), while no statistical difference was detected between treated and untreated CHIKV‑TEV (ns).

**Fig. 10:**
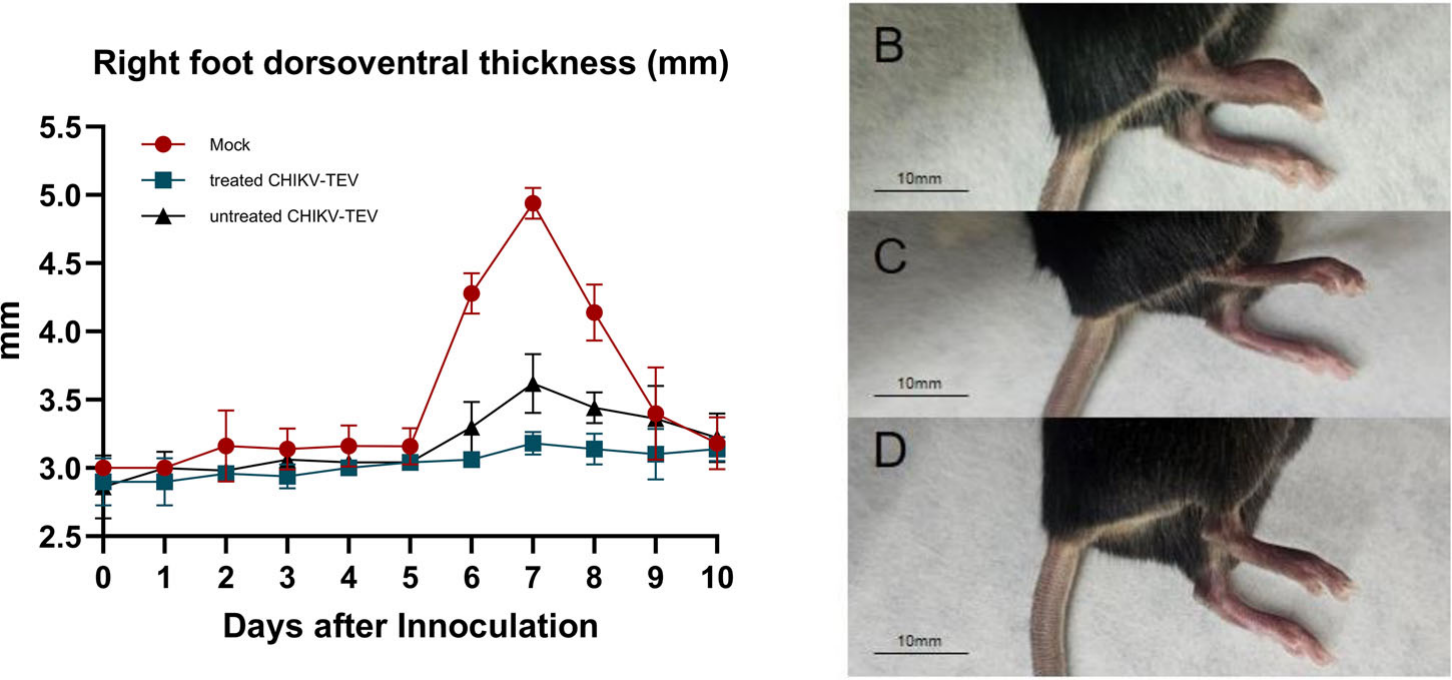
Right‑foot dorsoventral swelling in C57BL/6 mice following CHIKV infection (10 animals each group). **A** Time‑course plot of right‑foot dorsoventral thickness (in millimeters). Symbols indicate significant differences (*p* < 0.05): * between mock and treated CHIKV‑TEV, δ between mock and untreated CHIKV‑TEV, and φ between treated and untreated CHIKV‑TEV groups.). Representative images of right‑foot edema on day 7 for mock (**B**), treated CHIKV‑TEV (**C**), and untreated CHIKV‑TEV (**D**) mice, consistent with the quantitative measurements.

Mice inoculated in the right footpad with 10⁶ BzH1 particles developed a clear and time‑dependent swelling response over the 10‑day monitoring period (Fig. 10A). Mock‑infected controls showed a marked increase in dorsoventral thickness, which peaked around day 7 at approximately 5.0 mm. In contrast, both CHIKV‑TEV groups exhibited substantially reduced edema throughout the observation window. Swelling in the untreated CHIKV‑TEV group reached a peak of about 3.5 mm, while the treated CHIKV‑TEV group remained near baseline levels, showing only minimal variation over time. Significant differences were detected at the peak of inflammation (* between mock and treated CHIKV‑TEV; δ between mock and untreated CHIKV‑TEV; φ between treated and untreated CHIKV‑TEV). Representative images of each group on day 7 (Fig. 10B–D) illustrate the pronounced edema in mock controls and the attenuated swelling in both CHIKV‑TEV groups.

**Fig. 11:**
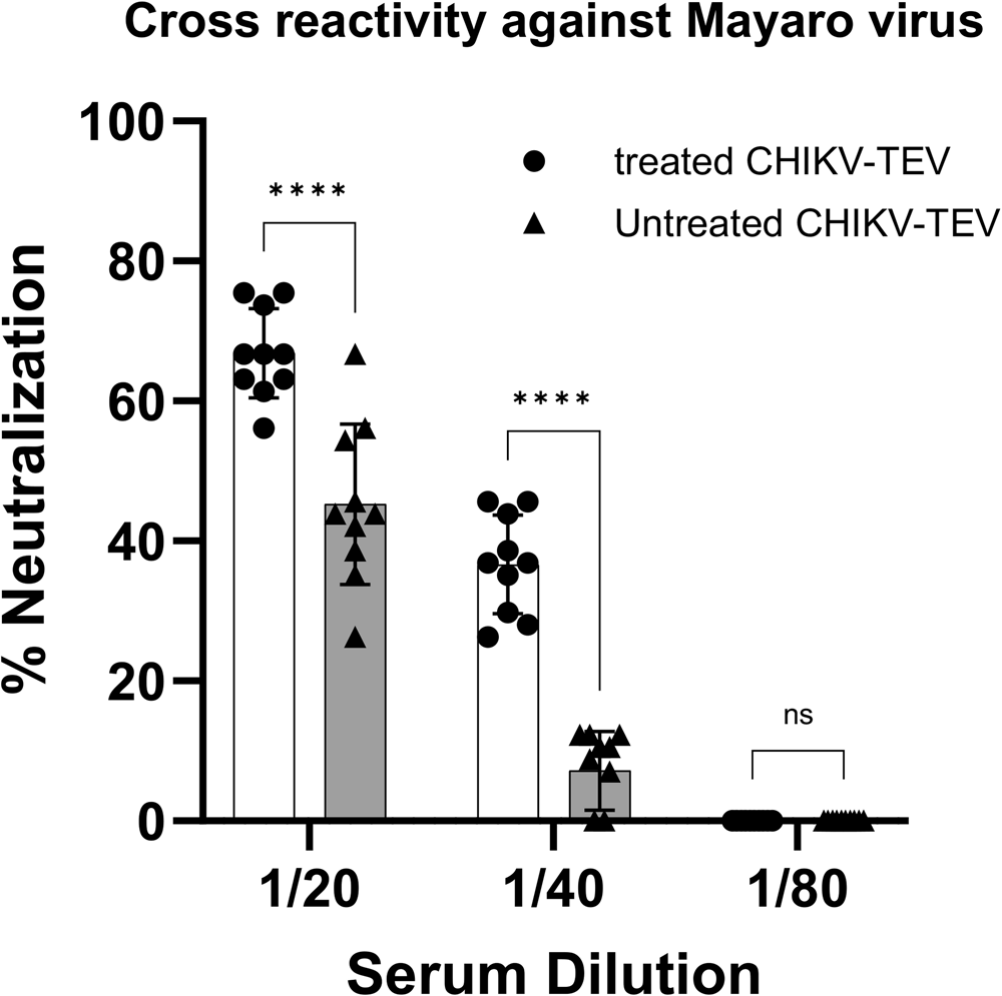
Cross‑neutralization of MAYV by sera from immunized C57BL/6 mice (10 animals each group), measured by plaque reduction neutralization assay. Neutralization was detectable only at the 1:20 and 1:40 dilutions, with the treated CHIKV‑TEV group showing significantly greater inhibition than the untreated group (^**^p < 0.001).

C57BL/6 mice serum was also tested for cross‑neutralization against Mayaro virus (MAYV). Only the two most concentrated serum dilutions (1:20 and 1:40) showed detectable neutralizing activity, indicating a modest cross‑reactive response. Within these dilutions, serum from mice immunized with treated CHIKV‑TEV particles consistently displayed higher levels of MAYV neutralization than the untreated CHIKV‑TEV group, mirroring the results observed for CHIKV neutralization (Fig. 11).

## Discussion

Since its introduction to the Americas between 2013 and 2014, the CHIKV has become endemic in the region. In recent years, transmission has increased, posing a growing challenge to public health. Brazil has been particularly affected, facing recurrent outbreaks and becoming one of the main hotspots for viral circulation. In 2023, the country reported over 158,000 suspected cases and 122 deaths, and in 2024, that number reached around 265,000 with 243 confirmed deaths^26^. Besides Brazil, CHIKV infections have been reported in other countries, including La Réunion, which experienced more than 47,500 cases, and Mayotte, where a resurgence of autochthonous cases has been observed^27^.

The development of safe and effective vaccines against CHIKV remains a major public health priority, especially given the expanding geographic distribution of the virus and the current lack of a vaccine that is safe and approved for use across all age groups. In this study, we propose an innovative vaccine platform based on non-infectious, immature CHIKV particles. These particles are genetically engineered to replace the native furin cleavage site with a TEV protease-dependent cleavage system, enabling precise control over viral maturation and enhancing the safety profile of the candidate vaccine.

Our data demonstrate that LoVo cells, which lack functional furin due to a truncating mutation, produce CHIKV particles that are structurally and functionally distinct from mature virions. Electron microscopy revealed that immature particles exhibit a significantly larger diameter (120.3 ± 21.45 nm) compared to mature particles (75.93 ± 11.74 nm), consistent with retention of the E3 glycoprotein, a hallmark of immature alphavirus particles^28^. This structural difference was further corroborated by infectivity assays, where immature particles showed delayed replication kinetics in furin-competent cells (Vero, HeLa, C6/36) but remained non-infectious in LoVo cells unless furin activity was restored via plasmid transfection.

In the absence of maturation, the resulting viral particles remain immature and are typically non-infectious. While most studies on immature particles have focused on dengue virus, little is known about the infectivity of immature alphavirus particles. The removal of the E3 protein is considered essential for the alphaviral particle to acquire infectivity, a process that usually occurs in trans-Golgi vesicles^24^, but may also take place in the endosome following particle internalization, through the action of furin^29^. Previous studies on Sindbis virus and other alphaviruses have demonstrated that furin-mediated cleavage of the p62 (E3–E2) precursor is critical for viral maturation and infectivity, and furin inhibitors or molecules that prevent vesicle acidification can affect the replication of these particles^18^.

The inability of immature particles to propagate in furin-deficient systems underscores the potential of targeting viral maturation as a strategy to inhibit replication and attenuate pathogenicity. To further study viral replication and the safety of our vaccine candidate, we replaced the furin cleavage site with a TEV protease recognition sequence, generating CHIKV-TEV particles. These particles were non-infectious in standard cell cultures but regained infectivity upon in vitro TEV treatment, confirming that proteolytic maturation is strictly required for viral entry and replication. Importantly, CHIKV-TEV particles did not produce plaques in Vero cells unless pre-treated with TEV protease, and their infectivity was not restored upon serial passage, eliminating concerns about reversion to virulence.

Our TEV-dependent maturation system offers a unique advantage: the ability to amplify non-infectious CHIKV-TEV particles, which can then be matured in vitro under controlled conditions before vaccination. This approach allows the virus to undergo a single round of replication in the host, ensuring the expression of non-structural proteins and the induction of a robust immune response. Importantly, all progeny particles remain non-infectious, serving solely as antigens to stimulate antibody production.

Although we have not measured the extent of in vitro cleavage of our construct, the results demonstrate that the system behaves as intended: viral particles treated in vitro with TEV protease were able to replicate in cells and generate virus progeny, and although these particles remained immature due to the absence of the furin‑cleavage site, they could still infect cells that express endogenous furin. Because TEV protease is not present in vertebrate or invertebrate hosts, TEV‑dependent particles are intrinsically unable to spread in natural environments. The absence of infectious virus after five sequential passages in both *Aedes albopictus* C6/36 cells and Vero cells further validated system safety. Given that the engineered construct lacks the native furin‑cleavage site and the TEV‑recognition sequence is distinct from the original cleavage site, reversion to a fully infectious phenotype is highly unlikely. In line with these observations, sequencing of viral progeny after multiple passages was not performed in this study because infectious particles were not recovered in the supernatant in the absence of TEV, preventing a direct assessment of genetic stability under non‑permissive conditions. Nonetheless, to fully characterize the evolutionary robustness of this platform, future studies will be needed to evaluate the genetic stability of the construct by sequencing the virus recovered from cells cultured in the presence of TEV.

In many viral infection models, vaccine efficacy can be evaluated by comparing clinical signs, virological parameters, or survival between vaccinated and naïve animals after challenge with a wild-type virus. For survival studies in particular, interferon receptor–deficient mice have been widely used as a susceptible model in arbovirus research, including dengue^30,31^, ZIKA^32^, and CHIKV^33,34^.

Despite being reported in the literature as inducing weak humoral and cellular immune responses^35,36^, the IFNAR^−/−^ mice used in the present study responded well to the vaccination protocol with CHIKV-TEV-treated particles, in a dose-dependent manner (the higher the viral load in the vaccine, the greater the survival rate). Although the challenge dose was relatively low (approximately 100 PFU of wild-type virus), the increased susceptibility of these mice, due to both their impaired interferon signaling and reduced antibody production, resulted in high mortality in the unvaccinated group, even at this low inoculum.

In this context, studies have shown that mosquito saliva contains high concentrations of CHIKV (around 10³ FFU/saliva)^37^. However, considering that only about 1 µL of saliva is injected during a mosquito bite^38^, the actual viral load transmitted by the vector is quite limited. Drawing a parallel to human infections, this suggests that even highly immunocompromised individuals (as occurs in the deficient mice used in the study) could be protected by the vaccine candidate tested here, as it effectively prevented disease even when challenged with virus doses similar to those delivered by natural mosquito transmission.

Although we did not directly measure the replication of TEV‑treated and untreated CHIKV‑TEV particles in vivo and therefore cannot formally correlate the in vitro replication data with in vivo viral kinetics, the robust protection conferred by TEV‑treated immature particles in IFNAR‑deficient mice, a highly susceptible model that could mimic severe CHIKV infection in immunocompromised individuals (e.g., elderly or immunosuppressed patients), demonstrates the safety and potential applicability of this vaccine candidate in vulnerable populations. This is particularly relevant in light of recent findings from the VALNEVA CHIKV vaccine (IXCHIQ®), a live-attenuated candidate that showed promising efficacy in phase 3 trials but was associated with adverse effects in older adults, including severe arthralgia and febrile reactions, leading to its recent suspension for individuals over 60 years of age. Unlike live-attenuated vaccines, which carry a risk of residual pathogenicity, especially in immunocompromised hosts, our immature particle platform is intrinsically non-replicating unless deliberately activated, offering a safer alternative for high-risk groups such as the elderly, immunocompromised individuals, and pregnant women.

Furthermore, we chose to immunize 3‑week‑old mice, a stage at which the murine immune system is functional but still maturing, to approximate early‑life immunity. Immunologic maturity in mice is gradually acquired over the first 3–5 weeks of life, and 3‑week‑old animals are considered a transitional stage analogous to the pediatric immune system in humans^39^. Thus, in addition to demonstrating safety and efficacy in a highly susceptible IFNAR‑deficient model that mimics severe disease in immunocompromised individuals, the robust protection observed in 3‑week‑old mice suggests that our vaccine candidate may also be suitable for use in younger populations, including children.

In the present study, the IFNAR^−/−^ mouse model was used mainly as an initial, more sensitive model to evaluate survival and lethality after CHIKV challenge, before moving to a wild-type model that better allows characterization of adaptive immune response. Although vaccine efficacy was demonstrated in IFNAR^−/−^ mice (as mentioned, a model widely used in immunoprotection studies), there is ongoing debate regarding whether an interferon‑deficient system is suitable for evaluating adaptive immunity. Therefore, a single immunization was performed in immunocompetent C57BL/6 mice to assess humoral protection, post‑challenge viremia, and the foot‑swelling model. Twenty‑one days after immunization with 10³ copies of TEV‑treated or untreated virus, the TEV‑cleaved group displayed a 9.14‑fold higher antibody titer compared with the untreated group, indicating that the initial round of replication enabled by TEV treatment acts as a signal amplifier for antibody production.

Neutralizing antibody titers have consistently been used as the primary indicator of vaccine‑induced protection for CHIKV. Clinical studies of the IXCHIQ® live‑attenuated vaccine demonstrated that high seroconversion rates and robust neutralizing responses were strongly associated with protection, with most protected individuals exhibiting PRNT₅₀ titers of ≥150. These thresholds align with earlier animal and human challenge data showing that even moderate neutralizing activity is sufficient to prevent viremia and clinical disease^40^.

In our study, the TEV‑treated group generated markedly higher antibody titers (PRNT_50_ > 686), suggesting that the limited replication enabled by TEV cleavage enhances the magnitude of the humoral response. Although the untreated group developed detectable neutralizing antibodies, the titers remained below the expected protective threshold^40^ (PRNT_50_ = 75), which is consistent with what is typically observed for single‑dose inactivated vaccines, a profile that resembles our non‑treated particle.

Sera from mice immunized with CHIKV‑TEV particles also neutralized MAYV, another alphavirus circulating in Brazil. This cross‑neutralizing activity likely reflects the antigenic similarity shared by viruses within the Semliki Forest complex. At serum dilutions of 1/20 and 1/40, both treated and untreated CHIKV‑TEV‑vaccinated groups showed significant MAYV neutralization compared with mock controls, with higher titers in animals receiving treated particles. Cross‑reactivity among alphaviruses has been reported previously, showing that antibodies generated against CHIKV can partially neutralize MAYV and related alphaviruses due to conserved epitopes in E1 and E2^41^. Similar patterns of serological cross‑neutralization have been described in experimental infections, human cohort studies, and vaccine‑based models, supporting the idea that humoral responses against CHIKV can extend to antigenically related alphaviruses^41–43^.

Cross‑reactive antibody response was evident but limited to MAYV, and, since the backbone for CHIKV-TEV constructs was from Mauritius strain (East/Central/South African genotype) and the challenge strain was from the Asian genotype (BzH1), we can infer that antibodies produced against our CHIKV-TEV construct can cross-neutralize other CHIKV strains and, to some extent, other alphaviruses from the same antigenic complex. Other studies have also reported antibody cross‑reactivity in both binding and neutralization assays using sera from individuals immunized with CHIKV vaccines (IXCHIQ and VIMKUNYA)^40,42^. These vaccine‑induced antibodies have the potential to recognize and neutralize antigenically related alphaviruses, suggesting that high antibody levels may provide a degree of cross‑protection within the Semliki Forest antigenic complex.

The analysis of footpad swelling after CHIKV challenge showed a clear protective effect in both vaccinated groups compared with mock‑infected animals. Mock mice developed a pronounced increase in dorsoventral foot thickness, peaking around day 7, consistent with the typical acute inflammatory response induced by CHIKV. In contrast, animals immunized with either treated or untreated CHIKV‑TEV particles exhibited a reduced or absent swelling throughout the course of infection. Although both vaccine groups were protected, mice immunized with the treated CHIKV‑TEV particles showed an even milder inflammatory response. The reduction in foot thickness relative to the untreated CHIKV‑TEV group was modest in absolute terms, but the difference reached statistical significance at the peak of edema (days 7 and 8), indicating that TEV treatment further improved control of local inflammation.

Immunization with the treated CHIKV‑TEV preparation produced a level of protection comparable to that reported in pre‑clinical studies using the attenuated IXCHIQ vaccine. Notably, even with a dose of only 10³ particles, our vaccine formulation achieved protection similar to that observed in animals immunized with 10⁴–10⁶ particles of the IXCHIQ strain^43^, indicating that TEV treatment enhanced vaccine potency enough to match the efficacy of substantially higher doses described for IXCHIQ.

Regarding the differences observed between the treated and untreated CHIKV‑TEV particles, both formulations provided protection against CHIKV‑induced footpad swelling, but the response was consistently stronger in animals immunized with the treated preparations. Because untreated particles are non‑replicating, their behavior is similar to that of VLP‑based or inactivated CHIKV vaccines, which typically require higher antigen doses or multi‑dose regimens to achieve full protection. Previous studies with CHIKV VLPs have shown that the reduction of inflammation after challenge is dose‑dependent, with higher doses showing more pronounced protection^44^. Thus, our results demonstrated that the immature CHIKV‑TEV particle, when processed with TEV protease prior to immunization, elicits a stronger immune response and provides efficient protection with a single dose.

Given that the TEV-cleaved construct undergoes a single round of replication, it ensures the intracellular synthesis of all viral non-structural proteins. Although T-cell responses were not measured in this study, this intracellular expression of viral proteins is known to promote efficient antigen processing and presentation via MHC class I pathways. Theoretically, this mechanism favors the priming of CD8⁺ T cells, offering a potential advantage over non-replicating platforms (such as VLPs or untreated particles) which rely primarily on exogenous antigen presentation. However, this remains speculative in the context of our work, as we did not directly quantify CHIKV-specific T-cell responses. Future investigations will be needed to define the magnitude, quality, and protective role of CD4⁺ and CD8⁺ T-cell responses induced by this vaccination strategy.

Furthermore, the same technology used in the manufacture of recombinant CHIKV particles with replacement of the furin site by TEV can be successfully used for other arboviruses that are also dependent on maturation by this protease, such as other alphaviruses and flaviviruses, and even for the new SARS-CoV-2, that has its infectivity strongly dependent on furin cleavage of the spike protein^45^.

In conclusion, this study demonstrated, based on functional assays, that regular immature CHIKV particles can initiate infection in furin-competent cells, in contrast to their behavior in furin-deficient cells, suggesting that these particles are not completely non-infectious as previously assumed. This infectivity relies on furin‑mediated cleavage within the endosome, suggesting that these particles can act as infectious units under physiological conditions.

In addition, the replacement of the furin cleavage site in the virus genome by another protease sequence (TEV) generates particles that do not produce a cytopathic effect in infected cells and are not able to produce virus progeny, thus being considered non-infectious. When subjected to in vitro maturation with recombinant TEV protease, these particles could infect cells and produce new particles. However, the progeny is composed essentially of immature particles that cannot reinfect cells. This process ensures that the virus that has undergone in vitro maturation can infect cells once and produce all structural and non-structural proteins, but no further infection can occur with this construct.

The extent of the immune response generated by this new virus construct could be of great importance as a vaccine candidate. It represents a new approach to virus attenuation, which depends on the external maturation of particles using the TEV protease and subsequently on the production of non-infectious particles. The advantage of using a single immunization dose with a lower particle input simplifies vaccine administration while still ensuring full protection in vaccinated animals. As observed in our experiments, the in vitro TEV-treated particles consistently showed superior performance compared with the untreated particles.

## Methods

### Cells and media

#### Virus production and stock titration

CHIKV strains used in this study were CHIKV BzH1 strain, of Asian lineage [isolated in Brazil (accession number KT581023)], obtained after infection of Vero cells (ATCC CCL-81), and CHIKV Mauritius (accession number FJ959103) derived from an infectious cDNA clone after transfection of BHK-21 cells (ATCC CCL-10)^25^, and Mayaro virus (BeAr 20290) used for cross-reactivity experiments. Immature particles were produced after infection of LoVo cells (deficient for furin protease - ATCC CCL-229) with the CHIKV BzH1 strain. The immature particles’ supernatants were collected after 10 h of infection and concentrated by ultracentrifugation (120,000 × *g* for 3 h, over a 20% sucrose solution). Virus stocks were titrated either by plaque assay in Vero cells using tenfold serial dilutions of virus stocks^46^ or genome quantification using RT-qPCR.

RT-qPCR assays were performed using Taqman^®^ Fast Virus 1-Step Master Mix (Thermo Fisher^®^), with primers and probes previously designed for CHIKV detection^47^.

#### Immature particles characterization and infectivity assays

After its production, immature and mature particles (produced in LoVo and Vero cells, respectively) were submitted to electron microscopy. The supernatant of infected cells was clarified by centrifugation, and particles were diluted in complete media according to their viral titer to a final concentration of 10^4^ copies/mL. An aliquot of 10 µL of each particle was loaded on top of a Formvar/Carbon-coated grid for 2 min. The unbound sample was then removed from the grid and particles were stained with 3% uranyl acetate for 1 min. Excess uranyl acetate was removed, and particles were analyzed under the electron microscope (JEOL JEM-100CX II).

To verify its infectivity potential, immature particles were compared to mature virus particles in vitro in different cell lines [Vero, LoVo, C6/36 (ATCC CRL-1660), and HeLa (ATCC CCL-2)], with a Multiplicity of Infection (M.O.I) of 0.1. After adsorption, the cell monolayer was washed three times with 1× PBS, and cell supernatant was collected at 6, 12, 24, and 48 h. Viral load was measured by plaque assay.

Due to its lack of furin expression, LoVo cells were transfected with a commercial furin cDNA ORF clone (Genscript OHu16791) to verify if CHIKV replication could be restored. Transfection was performed using liposomes (Lipofectamine® 2000 – Thermo Fisher Scientific) and 0.25 µg of the plasmid. Cells were harvested at 1, 2, 3, 5, 7, and 24 h after transfection, and furin expression was verified by Western Blot (anti-Furin antibody, sc-20801 - Santa Cruz Biotechnology). Transient transfection with an empty pcDNA 3.1+ (Invitrogen) was performed as a negative control. Infections with immature particles were made after 8 h of transfection and viral load was measured by RT-qPCR 24 h post infection.

#### CHIKV Fur^−/−^ infectious clone construction and production of recombinant particles

The construction of the CHIKV infectious clones with replacement of the furin cleavage site was performed using a previous cDNA clone as a template, as described by Kümmerer, et al.^25^. Briefly, the cDNA clone template was produced by PCR fragments with specific primers (sequence available on request) designed to amplify virus genome fragments, which were assembled from overlapping restriction fragments in a propagation vector (pSMART-LCKan). Four different plasmids were constructed, two with replacement of the furin cleavage site with a TEV sequence (with and without mCherry signal), and the other two plasmids with furin cleavage site mutation (with and without mCherry signal) (GenBank accession number PX939275).

To replace the sequence encoding the furin cleavage site (RQRR) with the TEV cleavage site sequence (ENLYFQG), a small fragment of the virus sequence was synthesized (2226 base pairs) with the new cleavage site in a pUC57 plasmid (Genescript® - A0791K) flanked by two enzymes with single restriction sites (BglI and SgarI). The fragment was inserted into the original infectious clone plasmid and transformed into *Escherichia coli* bacteria. Colonies were selected and analyzed by PCR to verify the presence of a TEV cleavage site (Primers: F-5′-ACCTGTACTTCCAAGGCAGC-3′; R-5′CACCGTCAGAGTTTCCCCT-3′) and submitted to sequencing to verify sequence and frame of gene expression. For plasmids with a mutation in the furin site, we performed a point-specific mutation (Quickchange protocol), using the Phusion enzyme (ThermoFisher^®^), to replace the original furin cleavage site RQRR by SQRS, which could not be recognized by the furin protease. All final plasmids were amplified in bacteria and purified using a Midiprep kit (NucleoBond Xtra Midi kit, Macheret-Nagel^®^) following the manufacturer’s protocol, and sequenced to confirm correct plasmid identity.

All plasmids were linearized using NotI enzyme (New England Biolabs®), and a total of 1 µg was subjected to in vitro RNA transcription using a T7 enzyme (mMESSAGE mMACHINE T7 Transcription Kit, ThermoFisher^®^).

Transcripts were electroporated in BHK-21 cells, and were maintained in MEM (Minimum Essential Medium Eagle, Gibco^®^) medium supplemented with 7.5% FBS (Fetal Bovine Serum) and 1% L-Glutamine. A total of 10^6^ cells were subjected to electroporation (per transcript) (GenePulser XcellTM Electroporation Systems, BioRad^®^) using a pulse of 140 V and 25.0 mS in a 2 mm cuvette. A negative control cell was also electroporated without a transcript to verify cell survival. Electroporated cells were seeded in a 48-well plate in complete MEM and incubated at 37 °C with 5% CO_2_. Virus production and immunofluorescence for the mCherry signal were analyzed after 9, 24, and 30 h of electroporation.

After electroporation, viral titers were determined by plaque assay. Plaque assay was performed on Vero cells, in a 24-well plate with a confluence of cell monolayer (5 × 10^4^ cells per well, in MEM, supplemented with 7.5% Fetal Bovine Serum (FBS) and 1% L-Glutamine). Serial (10-fold) dilutions of the virus were seeded on cells and incubated for 60 min to allow adsorption of the virus, and a semi-solid medium (1.5% carboxymethylcellulose in MEM) was added to the cells. Cells were incubated for 48 h at 37 °C, with 5% CO_2_. The medium was removed, the cells were fixed with 6% formaldehyde buffer and then stained for 15 min in 1% crystal violet in ethanol solution. Plaques were counted, and virus stock was calculated in PFU (plaque-forming units) per mL.

Since only the positive controls (CHIKV wild type with and without mCherry) formed plaques, we performed viral RNA quantification (RT-qPCR) for all constructs after ultracentrifugation of the stock in a 20% sucrose cushion in 1X PBS for 5 h at 100,000 G (Beckman Coulter® Ultracentrifuge, SW40 Ti rotor) to remove the remaining RNA from the electroporation. The supernatant was discarded, and the virus pellet was resuspended in a complete MEM medium, aliquoted, and kept at -80 °C. An aliquot was subjected to RT-qPCR on a LightCycler 480 II Thermocycler (Roche®) using a one-step amplification kit (TaqMan® Fast Virus 1-Step Master Mix, ThermoFisher®), and virus titer was considered as the number of RNA copies per mL (viral load). Western Blot of virus stocks was also performed, using an anti-capsid protein (structural protein) antibody produced in rabbit (kindly provided by Andres Merits, University of Tartu, Estonia), to assure that particles were produced, and the titer was not due to remaining transfected RNA.

#### Treatment of TEV protease and cell infection

The produced CHIKV-TEV mCherry particles were incubated with TEV protease (Protean^®^) at 500 protease units to 2 × 10^4^ virus particles for 1 h at 30 °C and placed at 4 °C for a further 12 h (TEV-treated virus). Another aliquot of the CHIKV-TEV virus was subjected to the same procedure without protease (untreated TEV virus). Both the treated and untreated particles were purified by ultracentrifugation through a 20% sucrose cushion to remove any residual TEV protease before the experiments. As a positive control, wild-type CHIKV mCherry particles were also subjected to incubations (without TEV protease incubation). Viruses were then incubated for 1 h at 37 °C with BHK-21 cells in a 48-well plate or on an 8-well ibiTreat slide (Ibidi® Integrated BioDiagnostic) with an M.O.I. of 1 (10^4^ cells and 10^4^ viruses) in experimental replicates. After adsorption, cells were washed three times with 1X PBS, and a complete medium was added to the cells (350 µL).

The supernatant was collected after adsorption (T0) as a control and 24 h after the first infection (T24), clarified by centrifugation and used to infect new cells (re-infection – 250 µL of initial infection) to test whether the viral progeny could be infectious. The supernatant from both infections was subjected to RNA extraction (Nucleospin Virus RNA - Macherey-Nagel^®^) and RT-qPCR and cells were fixed for immunofluorescence.

Cells subjected to infection and reinfection were fixed with 6% formaldehyde for 30 min, permeabilized with 0.1% Triton X-100 in 1× PBS at 37 °C and incubated with anti-nsP1 CHIKV antibody (produced against purified nsP1 protein – kindly provided by Andres Merits), followed by secondary anti-rabbit antibody labeled with Alexa 488. The presence of the nsP1 protein was analyzed by immunofluorescence in a LED fluorescence microscope (Leica DFC 3000G, CoolLED pE 300 lite) (green fluorescence for nsp1 – Alexa 448 and red fluorescence for mCherry signal).

To assess whether the mutated virus, with or without prior TEV protease treatment, could recover its replication competence, five sequential 24‑h passages were performed. At each passage, the complete supernatant from the preceding infection was transferred onto fresh C6/36 and Vero cell monolayers, and the supernatant from each passage was quantified by plaque assay.

#### Immunization of mice with immature particles

CHIKV-TEV particles produced were evaluated for their use as a vaccine after in vitro cleavage of the E3 protein with the TEV protease. Three groups (10 animals each, per dose) of interferon (alpha/beta) receptor-deficient mice (IFNAR^−/−^ C57BL-6 background), all female and 3 weeks old, were immunized: a control group receiving PBS; a group that received untreated CHIKV-TEV particles at three different doses (10⁴, 10³, and 10² particles); and a group immunized with treated CHIKV-TEV particles at the same doses. Only one vaccination dose was administered per group per dose. All immunizations were performed via intraperitoneal injection in a 50 µL total volume. Mice were briefly anesthetized with Ketamine (80 mg/kg) and Xylazine (10 mg/kg) administered intraperitoneally to minimize stress and discomfort. After 28 days, an intraperitoneal challenge with 10 LD₅₀ of wild-type virus (BzH1 strain) was administered. Prior to challenge, animals were anesthetized with Ketamine (80 mg/kg) and Xylazine (10 mg/kg) administered intraperitoneally to ensure humane handling. The animals were monitored for 21 days, during which deaths and clinical signs of infection were recorded. Animals were housed under specific conditions (maintained at 22 ± 2 °C with 50–60% humidity and a 12-h light/dark cycle). Food and water were available *ad libitum*. To reduce experiment bias, all animals were randomly assigned to groups. Humane endpoints were established, and animals that did not succumb to infection were euthanized promptly after experiments. Euthanasia was conducted by intraperitoneal administration of an overdose of Ketamine (300 mg/kg) and Xylazine (30 mg/kg), according to recommended guidelines (CONCEA – National Commission for Ethics in Animal Experimentation in Brazil).

Wild-type C57BL/6 mice were immunized with treated or untreated CHIKV‑TEV particles. The vaccination protocol consisted of a single dose containing 10³ particles per animal (three groups: mock, treated CHIKV‑TEV, and untreated CHIKV‑TEV; thirty animals each). Immunizations were performed subcutaneously under ketamine (80 mg/kg) and xylazine (10 mg/kg) anesthesia. After 21 days, the animals in each group were distributed further into three experimental groups. The first group (10 animals each) was used for blood collection by cardiac puncture under anesthesia, following CONCEA guidelines; serum samples were processed for plaque reduction neutralization assays against CHIKV BzH1 and MAYV (BeAr 20290). The second group (10 animals each) was challenged subcutaneously with 10⁶ copies of wild‑type CHIKV (BzH1), and blood was collected 2 days later to quantify viremia by RT‑qPCR. The third group (10 animals each) received 10⁶ BzH1 particles in the right footpad and was monitored for 10 days to assess footpad swelling. Measurements were taken using a manual caliper on the dorsoventral thickness of the footpad.

All animal procedures were approved by the University of Sao Paulo Ethics Committee on Animal Use (CEUA-FMRP, protocol number 209/2017) and were conducted in accordance with relevant Brazilian national guidelines and international standards for the care and use of laboratory animals. No animals were excluded from the study.

### Statistical analyses

All statistical analyzes were performed using GraphPad Prism version 10.0 (GraphPad Software, San Diego, CA, USA). Data are presented as mean ± standard deviation (SD) from three independent experimental replicates. For comparison between two groups, an unpaired two-tailed Student’s *t*-test was used. When comparing more than two groups, one-way ANOVA was applied followed by pairwise comparisons (Tukey or Sidak, as indicated). A *p*-value < 0.05 was considered statistically significant.

## Supplementary information

Supplementary Figures

ARRIVE Compliance Questionnaire

## Data Availability

The datasets generated and analyzed in this study are available as supplementary material. CHIKV-TEV clone sequence is deposited in Genbank under accession number PX939275.
