# Supplementary material for: Maturation-deficient chikungunya virus elicits protective immune responses in a murine challenge model
Source: NPJ Vaccines. 2026 May 8;11:98. doi: 10.1038/s41541-026-01478-w (PMC13153275; doi:10.1038/s41541-026-01478-w)
Supplement: Supplementary file 1 — Supplementary Figures [file 41541_2026_1478_MOESM1_ESM.pdf]

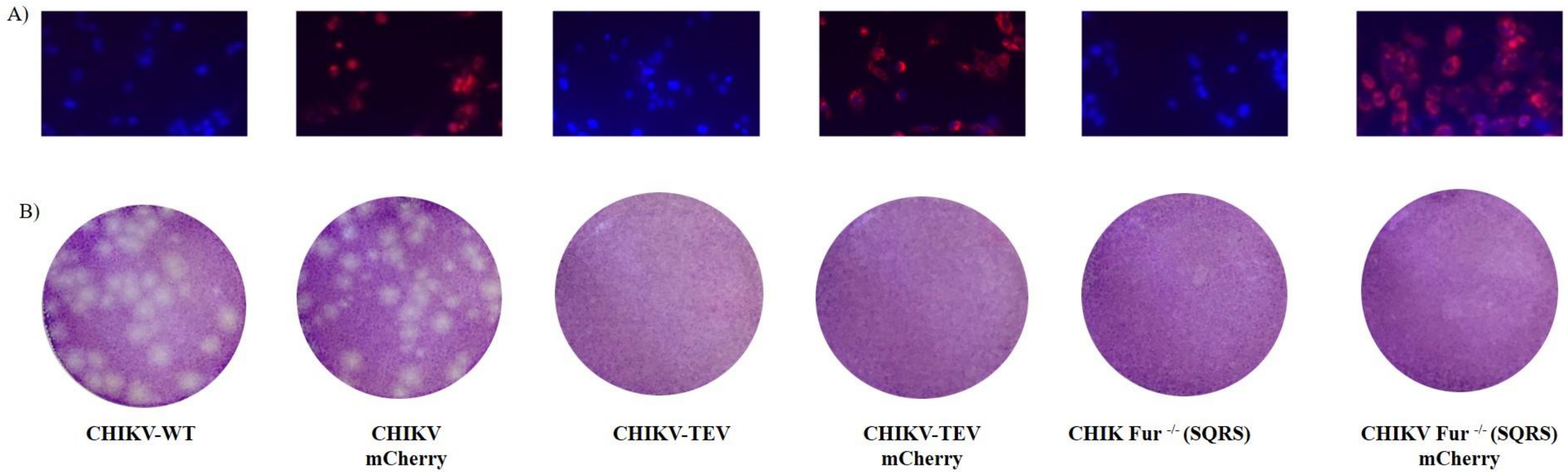

Supplementary Figure 1 – Evaluation of reporter expression and plaque formation of CHIKV constructs. (A) Representative fluorescence microscopy images of BHK-21 cells at 24 hours post-electroporation, showing mCherry signal (red) and nuclei counterstained by DAPI (blue). (B) Infectious center assay of CHIKV-WT, CHIKV mCherry, CHIKV-TEV, CHIKV-TEV mCherry, CHIKV Fur$^{-/-}$ (SQRS), and CHIKV Fur$^{-/-}$ (SQRS) mCherry, illustrating differences in plaque formation associated with viral replication and cytopathic effect. After electroporation of BHK-21 cells with the respective *in vitro* transcribed RNAs, electroporated cells were serially diluted and seeded together with feeder cells before adding an agarose overlay. At two days post-electroporation, cells were fixed and crystal violet stained. For the non-cytopathogenic viruses, the wells with the lowest dilution are shown, while for cytopathic viruses a higher dilution is represented, which allows individual plaques to be displayed.

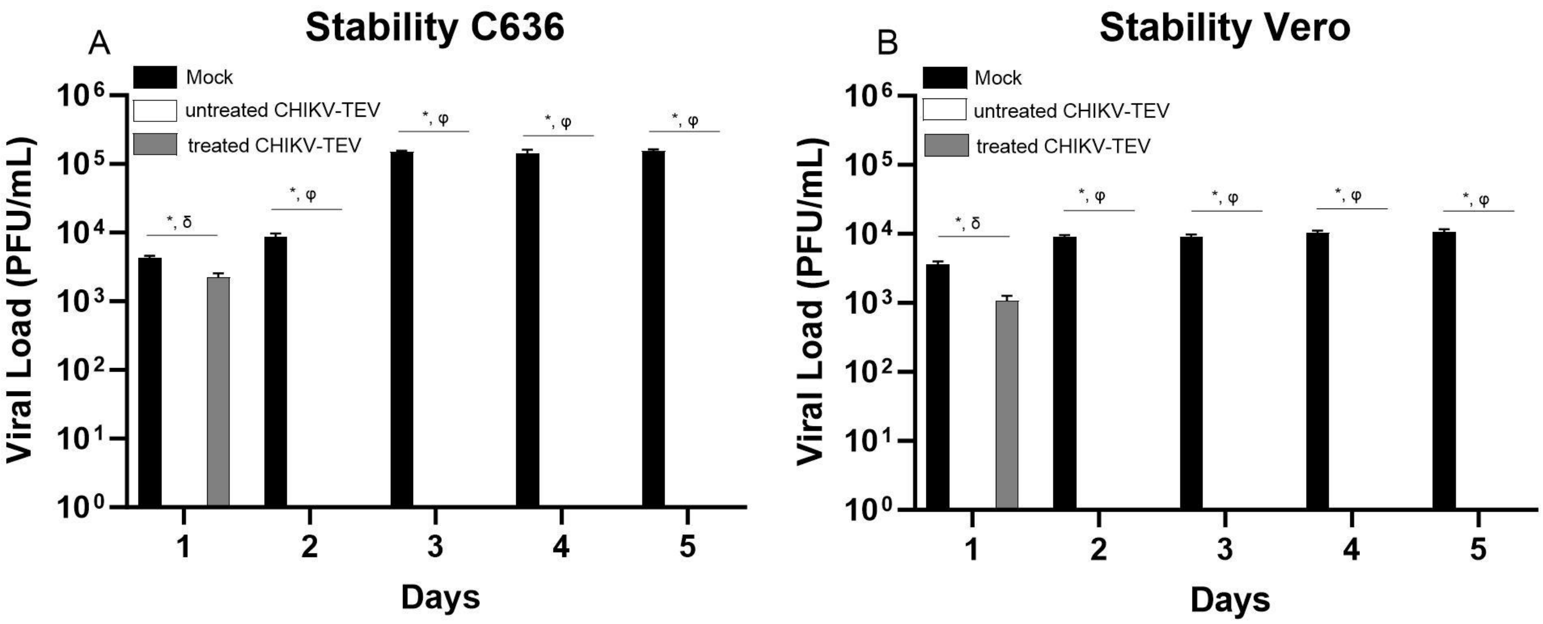

Supplementary Figure 2 - Stability of CHIKV-TEV constructs in C6/36 and Vero cells after sequential passages. Mutated virus, either untreated or previously exposed to TEV protease, was subjected to five sequential 24-hour passages. At each passage, the full supernatant was transferred to fresh C6/36 (A) or Vero (B) cell monolayers, and viral titers were quantified by plaque assay. Symbols indicate significant differences ($p < 0.05$): * between mock and treated CHIKV-TEV, δ between mock and untreated CHIKV-TEV, and φ between treated and untreated CHIKV-TEV groups. Statistical significance was assessed using Two-Way ANOVA test and Tukey's multiple comparison test.
